# Characterization of a Biofilm Bioreactor Designed for the Single-Step Production of Aerial Conidia and Oosporein by *Beauveria bassiana* PQ2

**DOI:** 10.3390/jof7080582

**Published:** 2021-07-21

**Authors:** Héctor Raziel Lara-Juache, José Guadalupe Ávila-Hernández, Luis Víctor Rodríguez-Durán, Mariela Ramona Michel, Jorge Enrique Wong-Paz, Diana Beatriz Muñiz-Márquez, Fabiola Veana, Mayra Aguilar-Zárate, Juan Alberto Ascacio-Valdés, Pedro Aguilar-Zárate

**Affiliations:** 1 Departamento de Ingenierías, Instituto Tecnológico de Ciudad Valles, Tecnológico Nacional de México, Carretera al Ingenio Plan de Ayala Km. 2, Colonia Vista Hermosa, Ciudad Valles, San Luis Potosí C.P. 79010, Mexico; 15690303@tecvalles.mx (H.R.L.-J.); mariela.michel@tecvalles.mx (M.R.M.); jorge.wong@tecvalles.mx (J.E.W.-P.); diana.marquez@tecvalles.mx (D.B.M.-M.); fabiola.veana@tecvalles.mx (F.V.); 2 Facultad de Estudios Profesionales Zona Huasteca, Universidad Autónoma de San Luis Potosí, Romualdo del Campo, No. 501, Rafael Curiel, Ciudad Valles, San Luis Potosí C.P. 79060, Mexico; jose94guada@hotmail.com; 3 Unidad Académica Multidisciplinaria Mante, Universidad Autónoma de Tamaulipas, E. Cárdenas González No. 1201, Jardín, Ciudad Mante, Tamaulipas C.P. 89840, Mexico; 4 Facultad de Ciencias Químicas, Universidad Autónoma de San Luis Potosí, Av. Dr. Manuel Nava 6, Zona Universitaria, San Luis Potosí, San Luis Potosí C.P. 78290, Mexico; mayra.aguilar@uaslp.mx; 5 Facultad de Ciencias Químicas, Universidad Autónoma de Coahuila, Boulevard Venustiano Carranza s/n, República Oriente, Saltillo, Coahuila C.P. 25280, Mexico; alberto_ascaciovaldes@uadec.edu.mx

**Keywords:** *Beauveria bassiana*, biological control, oosporein, spore production

## Abstract

*Beauveria bassiana* is an entomopathogenic fungus that is used for the biological control of different agricultural pest insects. *B. bassiana* is traditionally cultivated in submerged fermentation and solid-state fermentation systems to obtain secondary metabolites with antifungal activity and infective spores. This work presents the design and characterization of a new laboratory-scale biofilm bioreactor for the simultaneous production of oosporein and aerial conidia by *B. bassiana* PQ2. The reactor was built with materials available in a conventional laboratory. *K_L_a* was determined at different air flows (1.5–2.5 L/min) by two different methods in the liquid phase and in the exhaust gases. The obtained values showed that an air flow of 2.5 L/min is sufficient to ensure adequate aeration to produce aerial conidia and secondary metabolites by *B. bassiana*. Under the conditions studied, a concentration of 183 mg oosporein per liter and 1.24 × 10^9^ spores per gram of support was obtained at 168 h of culture. These results indicate that the biofilm bioreactor represents a viable alternative for the production of products for biological control from *B. bassiana*.

## 1. Introduction

The production of spores and metabolites, such as antibiotics, enzymes, and pigments from filamentous fungi, has taken on global importance for the biological control of pests and biotechnological purposes [1,2,3,4,5]. These metabolites have been produced using fermentation systems, such as submerged fermentation (SmF) and solid-state fermentation (SSF), which differ in the nature of their operating conditions [6,7,8,9]. Traditionally, both processes (SmF and SSF) are vital for industrial spore production. SmF is used for the production of biomass and mycelium that will be inoculated into the solid substrate in SSF [10]. 

*Beauveria bassiana* is an entomopathogen and endophyte fungi used as a biocontrol agent against pest insects as a spore formulation [3,11]. It also produces enzymes and secondary metabolites, such as bassianin, tenellin, beauvericin, bassiacridin, and oosporein; this last one has been characterized as a soluble red pigment with the formula C_14_H_10_O_8_, and there is great scientific interest in mass producing it for biotechnological applications [12,13]. 

The in vivo production of a reddish coloration has been reported in *Musca domestica* (L.) at the end of infection by *B*. *bassiana* attributed to oosporein [14] as the principal molecule responsible for immune host suppression [15]. This molecule has attracted attention for its antimicrobial activities against bacterial and fungal phytopathogens [13,16,17], but it also exhibits cytotoxic properties [12,13,18]. Conversely, it has been observed that the use of fermented crude extracts (raw secondary metabolites) of *B*. *bassiana* benefits plant growth, inhibits the development of diseases caused by plant pathogenic fungi, and contributes to the production of phenolic compounds [5]. Instead of the use of synthetic insecticides and fungicides, biological control using spores and even metabolites represent a safe and feasible alternative for the control of pests [19], but production systems should be developed to obtain quality products. Currently, biofilm reactors represent a formidable strategy that combines SmF and SSF in one apparatus for the production of spores, biomass, and secondary metabolites [20]. The attachment of aerial hyphae to the inert support and the release of secondary metabolites to the medium, similar to natural development, give a better yield and quality of conidia [21], while the inert support can be reused, making it simple, user friendly, and inexpensive [6]. However, in a bioreactor system, many parameters should be determined, one of the most important being the volumetric mass transfer coefficient (*K_L_a*), to establish the aeration efficiency during the aerobic bioprocess that depends on the shape, size, agitation speed, air flow rate, etc. of the reactor, but not on its volume [21,22]. The measurement of microbial growth is also a concern. Measuring microbial growth in solid fermentation is a difficult task due to the adhesion of the biomass to the solid support. Therefore, the measurement of CO_2_ production is a feasible method for its estimation [23].

The aims of the current study were as follows: to design and characterize a biofilm bioreactor for the production and recovery of aerial conidia and oosporein by *Beauveria bassiana* at lab scale and to evaluate the fungal growth by monitoring the CO_2_ production.

## 2. Materials and Methods

### 2.1. Fungal Strain and Media

The *Beauveria bassiana* PQ2 strain was obtained from the Food Analysis Laboratory, Instituto Tecnológico de Ciudad Valles, Ciudad Valles, San Luis Potosí, México. The fungal strain was cryopreserved using glycerol and skimmed milk at −20 °C and reactivated in potato-dextrose agar medium for seven days at 27 °C. 

### 2.2. Biofilm Reactor Setup

**Figure 1:**
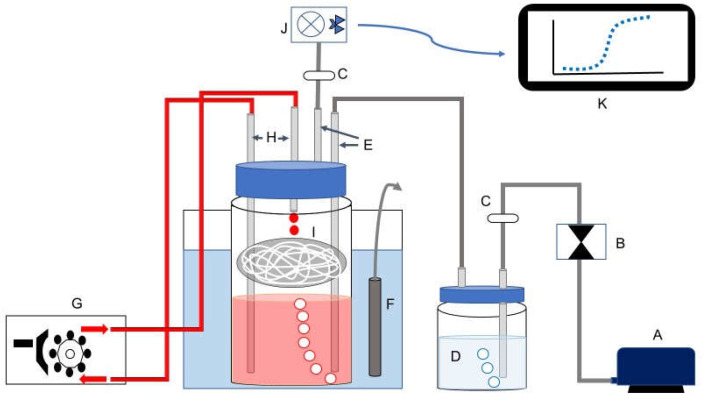
Scheme of biofilm bioreactor designed: (A) air pump; (B) air flow control; (C) air filter; (D) external bubbler; (E) air inlet and outlet; (F) thermostat; (G) peristaltic pump; (H) culture medium inlet and outlet; (I) inert support (metal structure packing); (J) wireless CO_2_ sensor; (K) LabQuest interface for data recovery. The samples were taken at the culture media inlet port.

The whole bioprocess was carried out in a low-cost lab-made biofilm reactor (Figure 1). The biofilm reactor consisted of a wide-mouth glass bottle with 1300 mL capacity (Schott Duran GLS 80, Mainz, Germany) and a 20 g stainless-steel pad (Scotch-Brite, 3 M, St. Paul, MN, USA) as inert support added to the bottle headspace (Figure 1). Air was supplied by an air pump. The medium was recirculated by a peristaltic pump (CRODE, Celaya, Mexico).

Volumetric oxygen transfer coefficients (*K_L_a*) were determined by three repetitions in both liquid and gaseous phases at three air flow rates (1.5, 2.0, and 2.5 L min^−1^) at 30 ± 2 °C. For the liquid phase, the sodium sulfite oxidation method was used [24]. A solution composed of 600 mL of Na_2_SO_3_ (0.5 N) and CuSO_4_ 0.001 M was used. The air inlet at different flow rates was started, and 2 mL samples from the solution were obtained in intervals of 1–8 h. To each sample, 3 mL of iodine (0.5 N) was added, and a titration with Na_2_S_2_O_3_ (0.06 N) was performed applying a starch solution (10%) as indicator. *K_L_a* was obtained using Equation (1):

(1) $$K_{L}a=\frac{1}{C^{*}}\times \frac{m\times N}{4\times Vm}.$$

where C* is the O_2_ solubility, *N* is the concentration of Na_2_SO_3_, *Vm* is the volume of sample of Na_2_SO_3_, and *m* is the slope obtained by linear regression (Figure S1).

For the gaseous phase, air flows of 1.5, 2.0, and 2.5 L min^−1^ (*F^in^_O_*_2_) were tested using the gaseous O_2_ sensor (Vernier, Beaverton, OR, USA) at the output flow (*F^out^_O_*_2_) in a solution composed of 600 mL of Na_2_SO_3_ (0.5 N) and CuSO_4_ (0.001 M) [25]. Samples from O_2_ were taken every 5 min for 480 min. After 480 min, the dissolved oxygen was determined by the Winkler method. The data obtained by the sensor and the Winkler method were used to obtain the gaseous phase balance with Equation (2):

(2) $$K_{L}a=\frac{F_{O2}^{in}-F_{O2}^{out}}{V\text{×}\left(C^{*}-C_{L}\right)}.$$

where *F^in^_O_*_2_ is the molar flow rate of oxygen gas input, *F^out^_O_*_2_ is the molar outflow of oxygen gas, *V* is the reactor volume, *C_L_* is the dissolved oxygen concentration in liquid phase, and C* is the oxygen saturation concentration.

### 2.3. Fungal Growth Evaluation, Aerial Conidia, and Red Pigment Production Dynamics

Submerged fermentation was carried out in two lab-made biofilm reactors (1300 mL) with 600 mL of Czapek–Dox mineral medium (sucrose 22.5 g/L, yeast extract 6.0 g/L, KH₂PO₄ 0.48 g/L, MgSO₄ 0.72 g/L, NH₄NO₃ 0.06 g/L, and CaCl₂0.24 g/L) at pH of 6.0. The culture medium was autoclaved at 121 °C for 15 min and inoculated with *Beauveria bassiana* PQ2 at 1 × 10^6^ spores/mL. Growth and pigment production were carried out in the following conditions: temperature of 30 ± 2 °C, air flow rate of 2.5 L/min, medium recirculation of 3.66 L/min (20 min, every 12 h), and duration of 168 h in the biofilm reactor for three repetitions. The oosporein and aerial conidia were monitored in the two bioreactors, while CO_2_ evolution data were obtained from only one bioreactor. 

**Figure 2:**
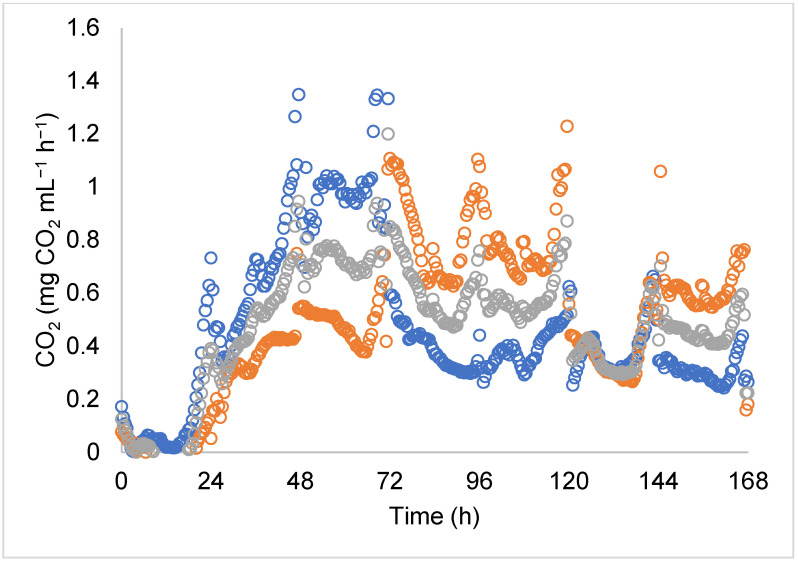
Evolution of CO_2_ production during fermentation of *Beauveria bassiana* PQ2 for the production of aerial conidia and oosporein. The different colors of open circles are the repetitions.

Microbial growth was estimated indirectly by CO_2_ production in the exit gases using a Go Direct^®^ CO_2_ analyzer (Vernier, Beaverton, OR, USA) controlled by LabQuest2 interface. The data generated show the CO_2_ production rate (Figure 2). The CO_2_ production rate was integrated to obtain the CO_2_ production. The CO_2_ production was modeled as biomass (mg CO_2_/mL of liquid media) by the Verlhurts–Pearl logistic model following the method proposed by Aguilar-Zárate et al. [26] (Equation (3)):

(3) $$\frac{dCO2}{dt}=\mu CO2\left[1-\frac{CO2}{CO2_{max}}\right]$$

where *µ* is the maximal specific CO_2_ production rate and *CO*_2_*_max_* is the equilibrium value for CO_2_ with *dCO*2/*dt* = 0. The solution to Equation (3) is shown as Equation (4):

(4) $$CO_{2}\left(t\right)=\frac{CO2_{max}}{1-\left(\frac{CO2_{max}-CO2_{0}}{CO2_{0}}\right)e^{-\mu t}}$$

*CO*2_0_ is the value of CO_2_ when *t* = 0. Square error values were minimized as a function of *CO*2*_max_*, *CO*2_0_, and *µ*.

Samples were taken every 24 h to the end of fermentation, and oosporein and sugar concentrations were measured. Sugar consumption was measured by refractometry since the sucrose was the carbon source. Oosporein analyses were carried out by spectrophotometry as follows. Samples were filtered through a sterile Millipore membrane (0.45 μm) (Minisart, Sartorius Stedim Biotech, Aubagne, France) and evaluated by spectrophotometer at the wavelength of 430 nm. Data were compared against a standard calibration curve of oosporein (0–31.25 ppm, purity ≥70%) provided by the Food Analysis Laboratory. The pigment production kinetics was modeled using the Luedeking–Piret model according to Aguilar-Zarate et al. [27] with Equation (5):

(5) $$\frac{d_{Oosp}}{dt}=Y_{Oosp/CO2}\frac{d_{CO2}}{d_{t}}+kCO2$$

where *Y_Oosp/CO_*_2_ is the production coefficient and *k* (mg/h mg) is the secondary coefficient of oosporein production (*k* > 0) or destruction (*k* < 0). The solution to the previous equation is given below (Equation (6)):

(6) $$Oosp=Oosp_{0}+Oosp_{Oosp/CO2}\;\left(CO2-CO2_{0}\right)+\frac{kCO2_{max}}{\mu }\;ln\;\left[\frac{CO2_{max}-CO2_{0}}{CO2-CO2_{0}}\right]$$

with *Oosp*_0_ being the value for oosporein when CO_2_ = CO2_0_.

The conidia production was evaluated at the end of the fermentation through the recovery from the metal solid support of the biofilm reactor with a diluted sterile 0.01% (*v*/*v*) Tween 80 solution. The fungal conidia were counted with a Neubauer chamber using a light microscope at 40×, and the spore yield was obtained by applying Equation (7):

(7) $$\frac{COnidia}{g\;of\;support}=\left(\frac{Spores}{mL}\right)\times \frac{Volume\;in\;suspension\;of\;conidia\;recovered}{g\;of\;support}$$

### 2.4. Oosporein Characterization by HPLC Tandem Mass Spectrometry

The filtered total aqueous extract at the final point of fermentation was analyzed by reversed-phase high-performance liquid chromatography (HPLC) equipped with an autosampler (Varian ProStar 410, Walnut Creek, CA, USA), ternary pump (Varian ProStar 230I, USA), and PDA detector (Varian ProStar 330, USA). A sample (5 μL) was injected into a Denali C-18 column (150 mm × 2.1 mm, 3.1 μm, Grace, Deerfield, IL, USA). The oven temperature was 30 °C. The elution gradient was formic acid (0.2 % *v*/*v*, solvent A) and acetonitrile (solvent B) with initial gradient course of 3% B, 5–15 min; 16% B linear, 15–45 min; and 50% B linear. The flow rate was 0.2 mL/min and the elution was monitored at 287 nm. Liquid chromatography–ion trap mass spectrometry (Varian 500-MS IT Mass Spectrometer, USA) equipped with an electrospray ion source was used. The MS analysis was performed in the negative mode [M-H]^−1^ using nitrogen as the nebulizing gas and helium as the damping gas. The parameters of the ion source were as follows: spray voltage of 5.0 kV, capillary voltage of 90.0 V, and temperature of 350 °C. Full scan spectra were acquired in the m/z range 100–2000, and, subsequently, the MS/MS analyses were performed on a series of selected ions. The data were collected and processed using MS Workstation software (V 6.9).

### 2.5. Statistical Analysis

The biofilm reactor setup experiments were performed with three repetitions, and the results are presented as mean ± SD. A post hoc analysis was carried out by Tukey test (*p* = 0.05) for comparing the significant differences between airflow rates.

## 3. Results

The determination of *K_L_a* by the sodium sulfite oxidation method for both liquid and gaseous phases allowed the characterization of the biofilm reactor. Table 1 shows that 2.5 L/min represents the best flow rate for aeration conditions in both solid and gaseous phases. 

**Figure 3:**
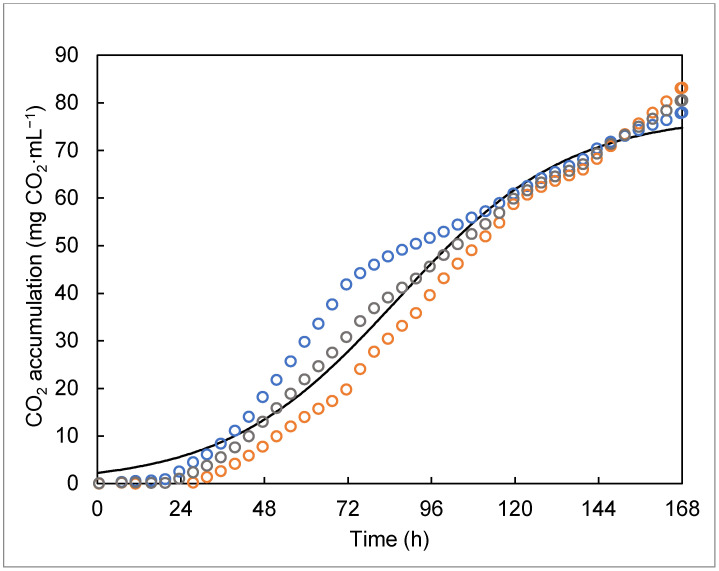
Accumulated CO_2_ production during fermentation of *Beauveria bassiana* PQ2 in the bioprocess for the production of aerial conidia and oosporein. Open circles are the experimental data, and the continuous line represents the data calculated by the model.

In regard to the respiration activity of *B*. *bassiana* throughout CO_2_ analysis, Figure 2 shows the evolution of CO_2_ production along the fermentation process. A similar pattern in CO_2_ evolution was seen during the 0–96 h period when submerged fermentation developed. High variation was observed during the 96–168 h period due to the high respiratory activity mainly in the solid-state culture. At 48 and 69 h, a higher CO_2_ production rate was observed (1.35 mg of CO_2_ per mL of media per hour). The maximal production rate was 80.59 mg CO_2_ mL^−1^ (Table 2) at 168 h of culture (Figure 3). It could be considered that the maximal growth rate was reached at this stage [6]. The accumulated CO_2_ is shown in Figure 3, and the data were obtained from the integration of the CO_2_ evolution. The graph shows the microbial growth trend at a growth rate of µ = 0.04 h^−1^ as follows: At 24 h, a lag phase was found as the entire fermentation process (liquid and solid) began biomass formation on the inert support at 50 h. Then, an exponential phase was observed followed by constant sugar consumption until the end of fermentation (168 h) (°Brix_initial_ = 4.03, °Brix_final_ = 0.55).

The production of aerial conidia did not present any problems using the metal structured packing as an inert support (Figure S2), allowing us to obtain 1.24 × 10^9^ conidia/gram of support at 168 h. Although part of the biomass was attached to the walls or internal parts of the biofilm bioreactor, its concentration was not considered in this study. The sterile conditions were confirmed, as there was no contamination of the culture medium. 

**Figure 4:**
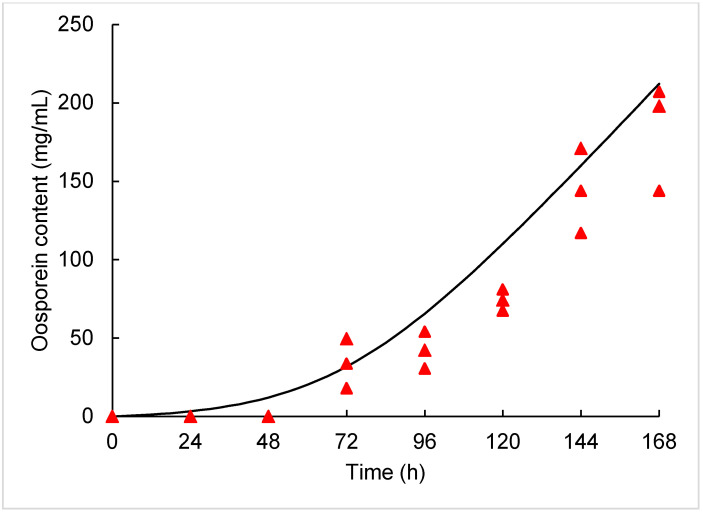
Kinetic production of oosporein by *Beauveria bassiana* PQ2 in biofilm bioreactor. Closed triangles are the experimental data, and the continuous line represents the data predicted by the model.

The production of the water-soluble pigment oosporein was achieved after 72 h (Figure 4), reaching a maximum concentration of 183 mg/L^−1^ at the end of the fermentation. The whole process reached an oosporein productivity of 1.09 mg/L/h (Table 2). The oosporein concentration increased over time, as shown in Figure 4. The production of oosporein reached two peaks associated with the growth in SmF and SSF. The first peak was obtained after 96 h of culture (42.30 mg/mL) (Figure 4). As shown in Figure 2, during 0–96 h, the submerged culture was developed. The second peak was obtained at the end of the fermentation process with 183 mg/mL of oosporein. The oosporein yield was 0.02 mg of oosporein per hour per mg of CO_2_.

**Figure 5:**
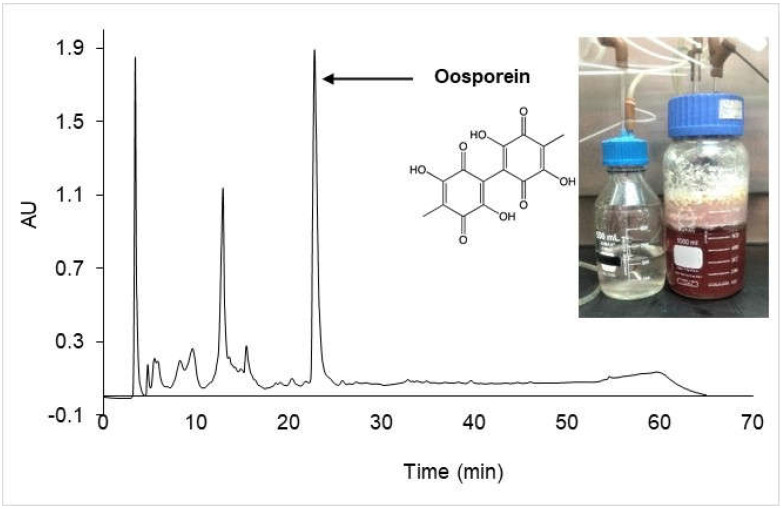
HPLC chromatogram of metabolites produced by *B. bassiana* PQ2 in the biofilm bioreactor.

Five hundred milliliters of fermented extract were recovered after 168 h and then submitted to an HPLC-MS/MS analysis in order to characterize the secondary metabolites and corroborate the presence of oosporein produced by *B. bassiana* PQ2. The results show the presence of five ionized compounds (Figure 5). The fragmentation patterns of the four compounds did not allow the identification of the molecules. Oosporein was found as the major compound in the HPLC chromatogram at a retention time (R. T.) of 22.72 min. It was identified with m/z 305 (306 M. W.) (Table 3).

## 4. Discussion

Biofilm reactors are a fermentation system for the production of value-added products that combine SmF and SSF approaches, where the biomass adheres to an inert support [28]. In this sense, the availability of oxygen in the solid fermentation (gaseous phase) will be exploited even when there is oxygen saturation in the liquid phase. In addition, the biomass attachment to the support confers some advantages, such as the low viscosity of the medium culture and easy downstream recovery of the products [29]. 

Oxygen availability is a critical factor for *B. bassiana* spore production [30,31,32,33]. The dissolved oxygen concentration is the result of the oxygen transfer rate (OTR) from the gaseous phase to the liquid phase and the oxygen uptake rate (OUR). The volumetric mass transfer coefficient (*K_L_a*) is a parameter that allows characterizing the capacity of a bioreactor to supply oxygen and is very important for the design, operation, and scaling of bioreactors [34]. Therefore, the *K_L_a* of the biofilm bioreactor was determined by two methods, one physical method and one chemical method. Under the operating conditions studied, a *K_L_a* value of 0.99 to 2.10 min^−1^ was obtained by the chemical method, while the physical method obtained values of 0.51 to 3.66 min^−1^. These values are close to those obtained for a circulating-bed biofilm reactor (0.017 s^−1^) [35] and higher than those obtained for an up-flow concurrent packed-bed biofilm reactor (0.0013–0.012 s^−1^) [36]. Furthermore, the *K_L_a* values obtained for the biofilm reactor built for this work are within the values (9.5–208 h^−1^) measured for different commercial stirred-tank bioreactors used for the production of *B bassiana* blastospores [37].

Hence, the bioprocess using *B. bassiana* PQ2 was carried out using the aeration flow rate of 2.5 L/min, which also contributed to the high availability of oxygen at the liquid–gas interface [38].

Respirometry analysis showed the metabolic activity of *B*. *bassiana* PQ2 when adapting to the fermentation conditions (Figure 2). From 48 to 69 h, CO_2_ production increased, which was related to mycelial development and the spore yield that improved after 100 h of the cultivation process [10,26]. However, the high variation of CO_2_ reported in Figure 2 might indicate the need to control the operating conditions, such as temperature, pH, and nutrients; thus, in the future, it will be necessary to optimize them [10]. Figure 3 shows that the microbial growth data were analyzed by non-linear regression. Based on the analysis, the growth rate (μ) was considered as the parameter to prove that the samples came from the same population. We obtained μ = 0.040 with a 95% of confidence interval of 0.039–0.042 and standard error of 0.001. The results shown in Figure 3 are similar to those reported by Cruz-Barrera et al. [9] for *Trichoderma asperellum* Th204 during SSF, who found that the CO_2_ accumulation indicated the lag phase occurred from 0 to 20 h, the exponential phase from 20 to 80 h, and the stationary phase from 100 to 160 h. Similarly, in the present work, CO_2_ production was observed even at the end of the process, as the microorganisms continued to grow on the inert support, and it is an indicator of metabolic activity of *B*. *bassiana* mycelium [39]. Some authors have reported the respiratory activity as an indirect indicator of fungal growth from *Aspergillus niger* GH1 [26], *Trichoderma harzianum* IRDT22C [26], *Metarhizium anisopliae* strain CP-OAX [10], and *Metarhizium anisopliae* IBCB 425 [40], but there is no information available for *Beauveria bassiana*.

In the biofilm bioreactor, the presence of conidiophores and spherical conidia attached to the biofilm agglomeration of mycelia on the metal solid support was found. Unlike aerial conidia, blastospores produced in submerged culture had an oval shape and formed fine pellets due to pneumatic agitation. At the end of the fermentation process, the mycelia produced under submerged fermentation were disintegrated, probably by the production of proteases. Once the fermentation process was finished (168 h), the aerial conidia were harvested from the metal solid support (Figure S2). The yield obtained was 1.24 × 10^9^ conidia/gram, which coincided with the reduction of CO_2_ production. This condition is similar to that reported by Méndez-González et al. [10] for spore production by *M*. *anisopliae* in SSF. In a solid-state culture, results close to those obtained in this study using *B*. *bassiana* were reported by Kang et al. [30] using a packed-bed bioreactor. They achieved 1.1–1.2 × 10^10^ g^−1^ and 1× 10^9^ conidia/g on grain substrates [41] and 5.0 × 10^8^ spores g^−1^ dry matter using rice husk [42]. These results serve as a comparison for the yield achieved from *B*. *bassiana* in SSF, indicating the biofilm bioreactor is a feasible tool for the production of conidia. 

The pigment production is consistent with that reported by Ávila Hernández et al. [43], who mentioned that oosporein is the major pigment produced by *Beauveria bassiana* under submerged fermentation, which shows antimicrobial and insecticidal activities. The yield obtained (183 mg/L^−1^) is close to the 270 mg/L^−1^ reported by Strasser et al. [44] from *B*. *brongniartii* in submerged culture. Amin et al. [45] produced red pigment from *B*. *bassiana* in submerged fermentation, reaching a yield of up to 480 mg/L. In addition, a combination of spores and pigment increases the insecticidal activity, which suggests that the use of the biofilm bioreactor developed in the present research would help to obtain infective units and metabolites in a single step for their possible use in the biological control of pests in further in vitro experiments. The production dynamics of oosporein (Figure 4) is similar to that of carbon dioxide (Figure 3) and may be another alternative for estimating fungal growth, because the use of certain metabolic products may offer more sensitive results [46]. 

The results obtained in the characterization of the compounds show five ionized peaks (Figure 5) where oosporein was the only relevant metabolite detected. It was identified by information reported in the literature, with the formula C_14_H_10_O_8_ [1,15,16,44]. The negative ionization of MS analysis allowed the identification of a compound with m/z 305. The result agrees with that reported by Feng et al. [15], who characterized the production of oosporein in fungi. They mentioned that oosporein is only ionized in negative mode. It was not possible to identify peaks 1-4, and other metabolites, such as tenellin, bassianin, or beauvericin produced by *Beauveria* species, were not found. Strasser et al. [44] considered that the production of oosporein is constitutive. Hence, *B. bassiana* PQ2 produced oosporein independently of the culture medium or the bioreactor. 

The results of this research represent a viable and novel way to produce aerial conidia and oosporein from *Beauveria bassiana* PQ2 using a biofilm reactor. Some authors have reported goods yields in protein production (hydrophobin II) by *Trichoderma reesei* [20]. In addition, conidia and secondary metabolites produced by *Aspergillus clavatus* in a biofilm reactor have been shown to be effective in mosquito (*Culex quinquefasciatus*) control [28]. 

## 5. Conclusions

The *K_L_a* of the biofilm bioreactor showed high oxygen transfer in both liquid and gaseous phases. The designed bioreactor ensured the development of SmF and SSF at the same time and the production of both aerial conidia and oosporein by *Beauveria bassiana* PQ2. The CO_2_ production dynamic allowed determining the fungal growth in the whole process. To our knowledge, this is the first report on the biomass estimation of *B*. *bassiana* through respirometry and the production of oosporein in a biofilm bioreactor. It represents a viable alternative for obtaining value-added products from filamentous fungi.

## Figures and Tables

**Table 1:** Bioreactor gas transfer in liquid and gaseous phases.

Flow Rate (L/min)	*K_L_a* Liquid Phase (min^−1^)	*K_L_a* Gaseous Phase (min^−1^)
1.5	0.99 ± 0.009 b	0.51 ± 0.261 b
2.0	1.16 ± 0.017 b	0.58 ± 0.102 b
2.5	2.10 ± 0.017 a	3.66 ± 0.394 a

Different letters indicate significant differences (Tukey test, *p* = 0.05).

**Table 2:** Kinetic parameters of *Beauveria bassiana* PQ2 under biofilm bioreactor conditions.

Parameters	Units	Value
CO_2 max_	mg CO_2_ mL^−1^	80.59
*µ*	h^−1^	0.04
Growth model	R^2^	0.99
Oosporein_max_	mgL^−1^	183.0
Oosporein productivity	mg/L/h	1.09
Oosporein model	R^2^	0.97
*Y_Oosp/CO_*_2_	mg Oosporein/h × mg CO_2_	0.02
Conidia recovery	Conidia/gram of support	1.24 × 10^9^

**Table 3:** Kinetic parameters of *Beauveria bassiana* PQ2 under biofilm bioreactor conditions.

Peak No.	R. T. (min)	M. W.	[M-H] ^−^ (m/z)	MS^2^ Ion Fragment	Tentative Identity
1	3.40	283	282	150, 133	Unknown
2	5.92	244	243	200, 110	Unknown
3	12.84	291	290	254, 230, 214, 200, 128	Unknown
4	15.49	387	386	343, 299, 298, 286	Unknown
5	22.72	306	305	277, 262, 261, 249, 233, 217, 205, 189, 161	Oosporein

R. T., retention time; M. W., mass weight; m/z, mass-to-charge ratio; MS^2^, tandem mass spectrometry.

## Supplementary Materials

The following are available online at https://www.mdpi.com/article/10.3390/jof7080582/s1, Figure S1: Evolution of oxygen solubility at different air flow-rates quantified by titration of liquid media with thiosulfate. Values of slope were used in Equation (1) for calculating Volumetric oxygen transfer coefficients (*K_L_a*) values, Figure S2: Invasion of metal solid support with mycelia and aerial conidia.
