# Supplementary material for: Characterization of a Biofilm Bioreactor Designed for the Single-Step Production of Aerial Conidia and Oosporein by Beauveria bassiana PQ2
Source: J Fungi (Basel). 2021 Jul 21;7(8):582. doi: 10.3390/jof7080582 (PMC8396940; doi:10.3390/jof7080582)
Supplement: Supplementary file 1 [file jof-07-00582-s001.zip › jof-1263914-supplementary.pdf]

Supplementary material

Characterization of a Biofilm Bioreactor Designed for the Single-Step Production of Aerial Conidia and Oosporein by *Beauveria bassiana* PQ2

Héctor Raziel Lara-Juache [1], José Guadalupe Ávila-Hernández [2], Luis Víctor Rodríguez-Durán [3,*], Mariela Ramona Michel [1], Jorge Enrique Wong-Paz [1], Diana Beatriz Muñiz-Márquez [1], Fabiola Veana [1], Mayra Aguilar-Zárate [4] Juan Alberto Ascacio-Valdés [5], and Pedro Aguilar-Zárate [1,*]

1 Departamento de Ingenierías. Instituto Tecnológico de Ciudad Valles, Tecnológico Nacional de México, Carretera al Ingenio Plan de Ayala Km. 2, Colonia Vista Hermosa, Ciudad Valles, San Luis Potosí, C.P. 79010, Mexico; 15690303@tecvalles.mx (H.R.L.-J.); mariela.michel@tecvalles.mx (M.R.M.); jorge.wong@tecvalles.mx (J.E.W.-P.); diana.marquez@tecvalles.mx (D.B.M.-M.); fabiola.veana@tecvalles.mx (F.V.)

2 Facultad de Estudios Profesionales Zona Huasteca, Universidad Autónoma de San Luis Potosí, Romualdo del Campo, No. 501, Rafael Curiel, Ciudad Valles, San Luis Potosí, C.P. 79060, Mexico; jose94guada@hotmail.com

3 Unidad Académica Multidisciplinaria Mante, Universidad Autónoma de Tamaulipas, E. Cárdenas González No. 1201, Jardín, Ciudad Mante, Tamaulipas, C.P. 89840, Mexico

4 Facultad de Ciencias Químicas, Universidad Autónoma de San Luis Potosí, Av. Dr. Manuel Nava 6, Zona Universitaria, San Luis Potosí, San Luis Potosí, CP. 78290, Mexico; mayra.aguilar@uaslp.mx

5 Facultad de Ciencias Químicas, Universidad Autónoma de Coahuila, Boulevard Venustiano Carranza s/n, República oriente, Saltillo, Coahuila, C. P. 25280, Mexico; alberto_ascaciovaldes@uadec.edu.mx

* Correspondence: luis.duran@docentes.uat.edu.mx (L.V.R.-D.), pedro.aguilar@tecvalles.mx (P.A.-Z.)

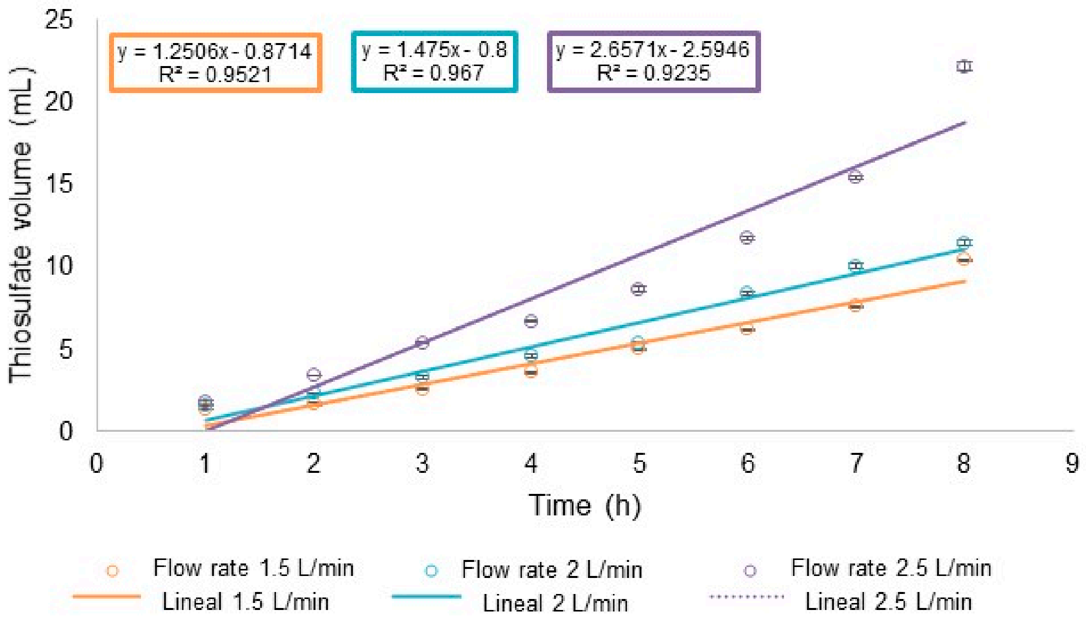

Figure S1. Evolution of oxygen solubility at different air flow-rates quantified by titration of liquid media with thiosulfate. Values of slope were used in equation 1 for calculating Volumetric oxygen transfer coefficients (K_La) values.

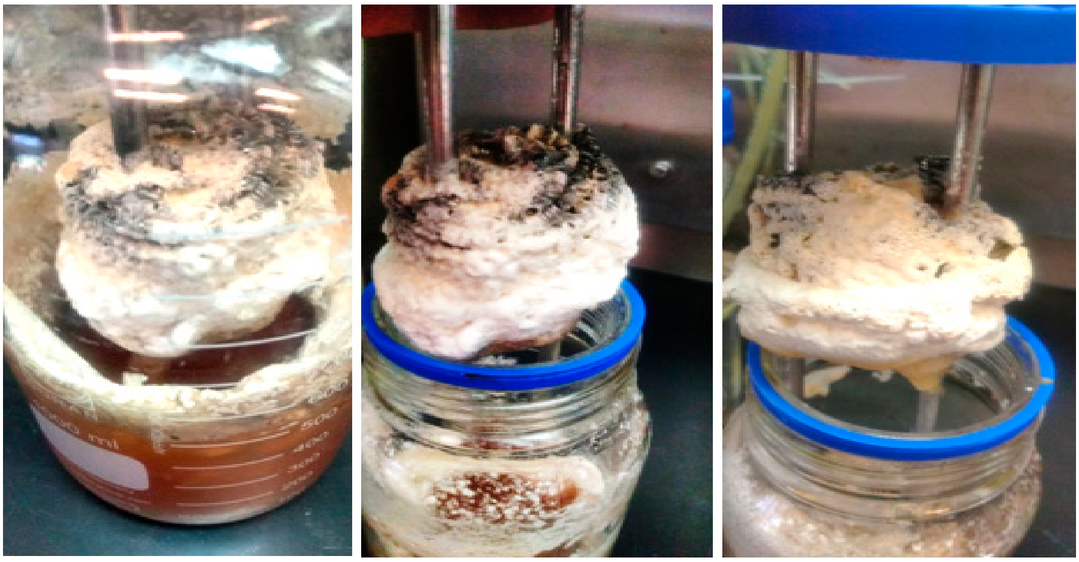

Figure S2. Invasion of metal solid support with mycelia and aerial conidia.
